# Enzyme disintegration with spatial resolution reveals different distributions of sludge extracellular polymer substances

**DOI:** 10.1186/s13068-016-0444-y

**Published:** 2016-02-03

**Authors:** Fan Lü, Jingwen Wang, Liming Shao, Pinjing He

**Affiliations:** State Key Laboratory of Pollution Control and Resources Reuse, Tongji University, 1239 Siping Road, Shanghai, 200092 People’s Republic of China; Institute of Waste Treatment and Reclamation, Tongji University, Shanghai, 200092 People’s Republic of China; Centre for the Technology Research and Training on Household Waste in Small Towns and Rural Area, Ministry of Housing and Urban-Rural Development (MOHURD) of China, Shanghai, People’s Republic of China

**Keywords:** Polygalacturonase, Polygalacturonic acid, Excitation and emission matrix, Environmental scanning electron microscope, Parallel factor analysis, Sludge flocs, Micro assembly

## Abstract

**Background:**

To understand the intrinsic role of hydrolytic enzymes in sludge treatment, particularly their effect on the digestibility and dewaterability of sludge, activated sludge flocs were disintegrated using various techniques that included different enzymes (amylase, cellulase, proteinase, DNase, and polygalacturonase), pH adjustment, and temperature adjustment. The effectiveness of each enzyme treatment was pinpointed by quantifying the spatial distribution of each type of organic matters (protein, polysaccharide, DNA, fluorescent organics) in outer layer extracellular polymeric substances (EPS), inner layer EPS, and cells.

**Results:**

Most hydrolytic enzymes functioned only owing to a temperature or pH effect. The release of organic matter from the interior fraction of EPS to the exterior fraction was prompted under high pH and temperature even without enzyme addition. The effectiveness of enzyme addition was only significant for cellulase and polygalacturonase treatments. Polygalacturonase unexpectedly increased the total EPS polysaccharides up to seven times, accompanied with improved dewaterability, while the amount of EPS proteins was almost unchanged. Combining chemical and morphological evidence, a new conceptual model considering the spatial distribution of polygalacturonic acid-like matter, proteins, cellulose, and other organics in EPS was proposed.

**Conclusions:**

Polygalacturonic acid-like matter hydrolysis caused significant release of polysaccharides. Polygalacturonase released polysaccharides while keeping proteins unreleased. Temperature and pH adjustment were as effective as enzyme at sludge disintegration. Cellulose hydrolysis led to massive release of all kinds of organic matters. A new conceptual sludge structure model regarding organic components is proposed.

**Electronic supplementary material:**

The online version of this article (doi:10.1186/s13068-016-0444-y) contains supplementary material, which is available to authorized users.

## Background

The dewaterability and digestibility of waste-activated sludge is of great importance in the treatment of this material. Various organic substances that comprise sludge, especially extracellular polymeric substances (EPS), are the well-known obstacles to digestion and dewatering [1], and pretreatment is often applied to overcome these obstacles [2–4]. Among the many pretreatment methods that aim at deconstructing EPS, enzymatic pretreatment stands out for economic, safety, and practical reasons. Because EPS is composed mainly of proteins and polysaccharides such as amylum and cellulose [5, 6], as well as minor fractions such as DNA and humic-like substance, hydrolytic enzymes targeting such polymers are frequently used in the pretreatment of sludge or for EPS extraction, e.g., protease [7–11], α-amylase [7, 8, 10–13], endogenous amylase [9], cellulase [8], lipase [11], pectinase [13], and DNase [13], individually, sequentially [13], or mixedly [11].

Unfortunately, there are still several unsolved problems in the current applications of enzymes for sludge treatment. For example, enzymatic treatments do not dramatically improve sludge digestibility. The choice of enzymes mainly has been usually limited to amylase and protease, so massive quantities of enzymes are needed to hydrolyze the proteins and polysaccharides that comprise the huge amount of EPS (200–500 mg/g volatile solids) in sludge [1]. The concomitant influences of temperature and pH adjustment usually have been neglected. Further, sludge treatment using only one enzyme usually yields unexpected results; to compensate for this, a complex mixture of enzymes is often used for better effect. However, the mechanism by which each enzyme in a mixture works is unknown, leaving the problem of how to decide the “recipe” of a complex enzyme mixture unsolved. Studies on hydrolyzing EPS lack specific details, such as what roles various types of organic matter have in EPS and what kind of enzyme can disintegrate EPS efficiently.

The present study was conducted to understand the intrinsic role of hydrolytic enzymes on EPS structure and consequently on the digestibility and dewaterability of sludge. Activated sludge flocs were disintegrated using different enzymes or using only pH adjustment or temperature adjustment. The effectiveness of each enzyme treatment on different organics was pinpointed by quantifying the spatial distribution of each type of organic matter (proteins, polysaccharides, DNA, fluorescent organics) in outer layer EPS, inner layer EPS, and cells. The application of fractionation approach was to make a better understanding on the roles of each organic matter on the flocculation of sludge aggregation by studying their distributions at different layers, which are partially important in sludge particles bridging and flocculating. In addition, principal component analysis was used to analyze the effect of different parameters on organic matters’ distribution. Environmental Scanning Electron Microscopy was used to visualize the morphology of sludge after different hydrolysis treatments. Special emphasis was placed on examining an unexpected polysaccharide-releasing, protein-conserving effect of polygalacturonase treatment. As a result, a new conceptual model describing the distribution of various organic compounds in EPS was developed.

## Methods

### Materials

The activated sludge used in this study was collected from a municipal wastewater treatment plant located at Shanghai (120°51′E, 30°40′N), treating 93 % municipal wastewater and 7 % industrial wastewater through anoxia–anaerobic–aerobic process. The sludge had a total solids (TS) content of 3 % wt and a volatile solids (VS) content of 68 % dw. The content (dry weight basis) of polysaccharides, proteins, and DNA content was 11.7, 37, and 3 % dw, respectively.

Five kinds of hydrolytic enzymes were used separately to specifically target polysaccharides, proteins, or DNA. Three enzymes were used to attack different polysaccharides: α-amylase (Sigma, Product No.: 10070-10G, about 50 U/mg derived from *Bacillus subtilis*, to be used at pH 6.9 and 25 °C), cellulase (Sigma, Product No.: C2605-50 mL, at least 1000 U/g derived from *Aspergillus sp.*, to be used at pH 8.0–8.2 and 40 °C), and polygalacturonase (Sigma, Product No.: 17389-10G, greater than 1 U/mg derived from *Aspergillus niger*, to be used at pH 4.0 and 50 °C). Proteins were targeted using proteinase (Yinggong Trading Co. Ltd., Shanghai, China, Product No.: LG1036, at least 200 units from *Bacillus licheniformis*, to be used at pH 6.0–8.5 and 50–60 °C). Lastly, DNA was attacked using DNase (Sigma, Product No.: DN25-10MG; at least 85% protein and at least 400 Kunitz units/mg protein derived from bovine pancreas, to be used at pH 7–8).

### Enzymatic treatment

Experiments were conducted in a constant-temperature incubator in magnetically stirred beakers that were sealed with waxed film. The dosages of enzymes, pH, and temperature for each treatment are listed in Table 1. The pH of the raw sludge was adjusted using hydrochloric acid and sodium hydroxide solutions to the optimal value (according to the producers’ suggestion and our preliminary trials) for each enzyme prior to enzyme addition. Since sludge digestion performs differently in acid, neutral, and alkaline environment, a control group of sludge samples without enzyme addition was prepared at each pH condition. Following pH adjustment and enzyme addition, the sludge samples were incubated at 35 or 55 °C. The optimal temperature for each enzyme we used is either around 35 or 55 °C. Furthermore, these are typical temperatures for mesophilic or thermophilic digestion. Control groups without enzymes were incubated under both temperatures, too. Gentle alkaline environment had been reported to promote sludge digestion but usually requiring long duration from 4 days to 20 days [14, 15]. Since we focus on enzymatic pre-treatments in this study, samples for analysis were collected 1, 2, and 5 h after incubation began, so that we could detect the changes as incubation continued and made sure the enzymes were still active. Each treatment was conducted in triplicate.

**Table 1:** Dosage of enzymes

Treatment no.	pH	Enzyme	Enzyme dosage (U/100 mL raw sludge)	Temperature (°C)
C-pH4-T35	4.0	Control	0	35
C-pH4-T55				55
C-pH7-T35	6.9	Control	0	35
C-pH7-T55				55
C-pH8-T35	8.0	Control	0	35
C-pH8-T55				55
Pec-pH4-T35	4.0	Polygalacturonase	420	35
Pec-pH4-T55				55
Amy-pH7-T35	6.9	α-Amylase	250	35
Amy-pH7-T55				55
Pro-pH7-T35	6.9	Proteinase	1000	35
Pro-pH7-T55				55
Cel-pH8-T35	8.0	Cellulase	500	35
Cel-pH8-T55				55
DNa-pH8-T35	8.0	DNase	1700	35
DNa-pH8-T55				55

### EPS extraction and stratification

Based on its strength of binding to cells, EPS was extracted and stratified through centrifugation and ultrasonication into four fractions: “supernatant,” “slime,” “loosely bound EPS” (LB-EPS) and “tightly bound EPS” (TB-EPS). The final pellet that remained after EPS extraction was mainly composed of microbial cells and unextractable EPS. The extraction procedure suggested by Yu et al. [16] was followed with some modification. An additional file describes the detailed procedure (see Additional file 1).

The aqueous suspended solutions of the enzymes themselves were stratified according to the same method, and every content was determined as sludge samples. Therefore, the reported values were subtracted from the enzyme background.

### Determination of organic components

The TS and VS contents of raw sludge were measured as the mass loss in samples following heating at 105 and 600 °C, respectively. The polysaccharides content was measured using Anthrone colorimetry [17] on sludge samples that were mixed with 50 mL 0.5 M H_2_SO_4_ and autoclaved at 121 °C for 1 h. Protein content was measured using the Lowry method [18] the same on sludge samples that were mixed with 50 mL 0.5 M H_2_SO_4_ and autoclaved at 121 °C for 1 h. DNA content was measured using diphenylamine colorimetry with 1 h incubation at 60 °C on sludge samples that had been subjected to two cycles of bead grinding at 6300 rpm for 2.5 min each.

After extraction, each EPS fraction was tested for polysaccharides, proteins, external DNA (eDNA), dissolved organic carbon (DOC), and dissolved nitrogen (DN). The determination of polysaccharides, protein, and eDNA followed the Anthrone colorimetry, Lowry method, and diphenylamine colorimetry, respectively. DOC and DN were analyzed using a Total Carbon/Total Nitrogen analyzer (TOC-V CPN, TNM-1, Shimadzu, Kyoto, Japan).

Raw sludge and pellets remaining after EPS extraction were first lyophilized and then tested for carbon, nitrogen, and hydrogen content using an element analyzer (Vario EL III, Elementar, Hanau, Germany).

### Fluorescent excitation–emission matrix analysis

Three-dimensional fluorescent excitation–emission matrix (EEM) analysis was performed using a Cary Eclipse Fluorescence Spectrophotometer (VARIAN, Santa Clara, CA, USA) to obtain the relative content of fluorescent organics in each fraction. The fluorescence was collected using an excitation wavelength of 200–550 nm and an emission wavelength of 250–600 nm. Parallel factor analysis (PARAFAC) was applied to the EEM data to qualitatively and quantitatively analyze the main fluorescent substances. The detailed procedure for EEM and PARAFAC analyses is given by Lü et al. [19].

### Environmental scanning electron microscopy (ESEM)

Environmental scanning electron microscopy (ESEM) was performed using a Quanta 250 (FEI, Hillsboro, OR, USA) to observe structural changes of biological samples due to enzyme treatment. Duplicate images of each sample were made at 500×, 1000×, 2000×, 5000× and 10,000× magnifications.

### Determination of sludge dewaterability

The dewaterability of sludge samples was evaluated according to the TS content of cakes obtained by centrifuging sludge at 2000×*g* for 10 min.

### Statistic analysis

In order to distinguish the effect of control group and experiment group at the same pH, analysis of variance (i.e., ANOVA analysis) was applied on the contents of polysaccharides, proteins, and eDNA in supernatant and TB-EPS fraction. pH 4.0 group includes pH 4.0 control and polygalacturonase-added group; pH 6.9 group includes pH 6.9 control, amylase-added group, and proteinase-added group; pH 8.0 group includes pH 8.0 control, cellulase-added group, and DNase-added group. Supernatant and TB-EPS are the two representatives of the four fractions.

To distinguish the significance of difference among samples after different treatments, PCA was applied. Four factors (the concentration of each category of organic matters in the supernatant, slime, LB-EPS, and TB-EPS fractions) were analyzed and converted to two principal uncorrelated variables, thus formed a map in which samples distributed according to their influence by two dimensions, and four factors’ contributions to principal variables were shown. The percentage of how much each principal component can explain the variance can also be calculated.

## Results and discussion

### Reallocation of different organic matters

#### Organic matters in the raw sludge

**Fig. 1:**
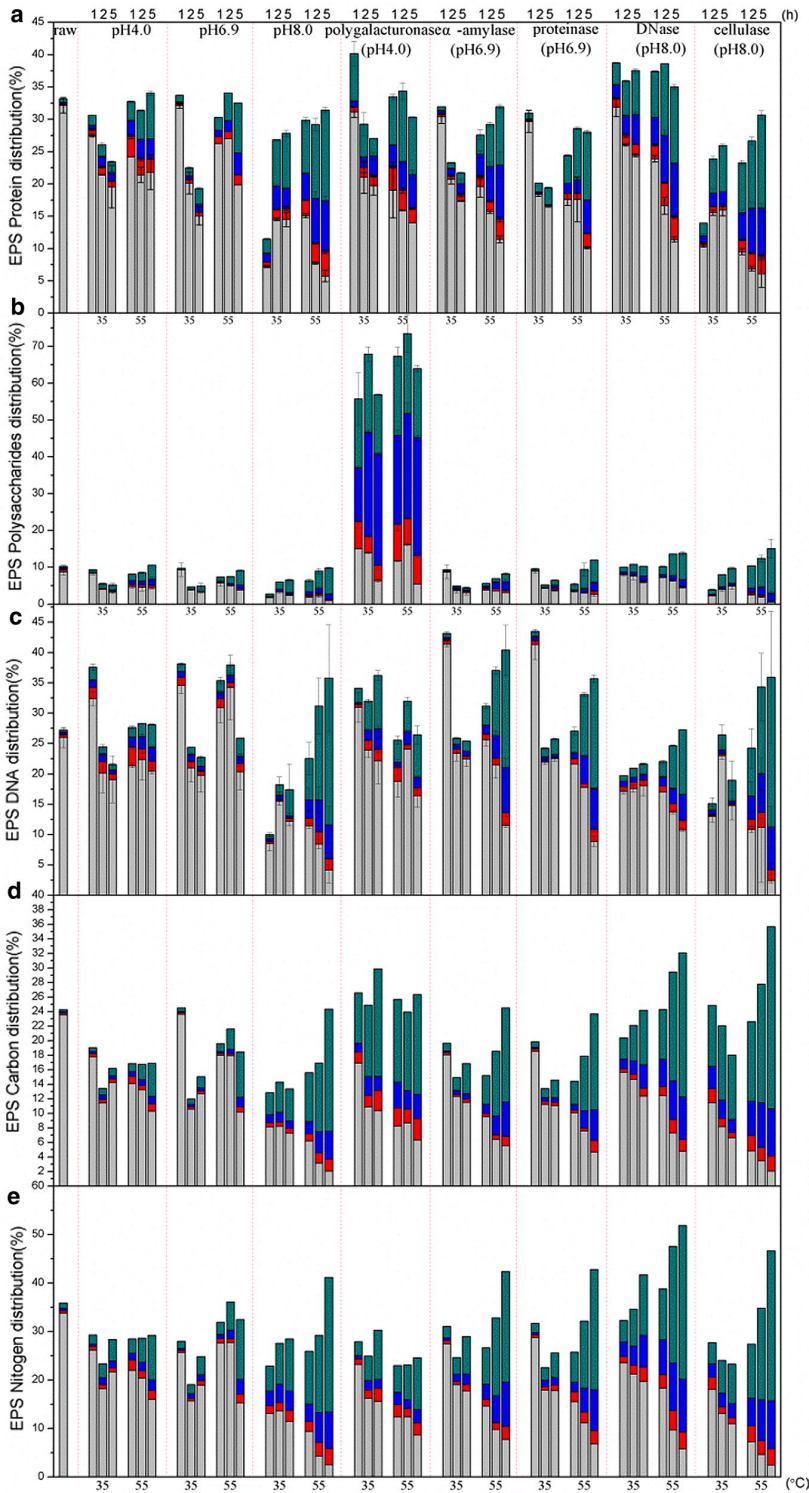
Distribution of organic matters in EPS out of sludge. **a** EPS protein; **b** EPS Polysaccharides; **c** EPS DNA; **d** EPS Carbon; **e** EPS Nitrogen. *Green square* supernatant; *blue square* slime; *red square* loosely bound; *gray square* tightly bound. The rest proportion belongs to pellet and potential loss during operation

Proteins, polysaccharides, and DNA comprised 70.5 % of sludge organics on a VS basis and were the dominant components in EPS. Figure 1a–c indicates the content of proteins, polysaccharides, and eDNA in each EPS fraction. To confirm the observed changes in organic substances that contained carbon or nitrogen, DOC (Fig. 1d) and DN (Fig. 1e) were assessed correspondingly. Raw sludge had 33.2 % of proteins, 10 % of polysaccharides, and 27.3 % of the eDNA in EPS, and of these, 97, 85, and 95.3%, respectively, were located in TB fraction. After treatments, the content in EPS was 11.5–40 % for proteins and 24–43.5 % for eDNA. For polysaccharides, the average total content of EPS was 2.7–15 %, but for the polygalacturonase treatment group, the EPS polysaccharide content was 55–73 %. And the location of each component significantly shifted from TB-EPS to LB-EPS, slime, and supernatant, in concern with the increase in treatment duration. Both DOC and DN followed the same trend. This may be owing to continuing enzymatic hydrolysis, sludge adsorption, and release of enzymes as they were still active.

#### Redistribution of proteins

The protein profiles after the various treatments exhibited three patterns: (1) in acid and neutral pH at 35 °C, the concentration of EPS proteins generally decreased as incubation time increased; (2) in alkaline pH at 35 °C, the concentration of EPS proteins firstly decreased significantly but then recovered as treatment time increased; and (3) in all pH at 55 °C, the total concentration of EPS proteins was unchanged, but redistribution of proteins occurred among fractions as the treatment duration increased. Contrary to expectations, the use of proteinase did not cause significant protein dissolution. Rather, compared to the pH 6.9 control treatment without enzyme addition, proteinase caused some shift of proteins from the TB-EPS fraction to supernatant only under the 55 °C treatment and caused no changes under the 35 °C treatment. The effects of amylase addition were similar to that of proteinase addition; however, amylase shifted more proteins to slime. The use of cellulase did not produce significant changes compared to the pH 8.0 control treatment. After incubation of samples with added polygalacturonase for 1 h at 35 °C, the proteins in supernatant were increased by ten times the content in raw sludge, but the supernatant content decreased as incubation duration increased, in agreement with the pH 4.0 control samples. The total amounts of EPS proteins in DNase-treated samples under both 35 and 55 °C treatments reached and remained at about 38 % ± 1.5 % of the proteins in sludge, and redistributions of these proteins occurred within EPS fractions. Noticeably, the decrease in the total amount of EPS proteins as incubation time increased in the alkaline treatment might have been caused by protein precipitation owing to denaturation and the assimilation of active microbes.

#### Redistribution of polysaccharides

The profile of EPS polysaccharides after the treatments followed four patterns: (1) in acid and neutral pH treatments at 35 °C, the concentration increased slightly at first but then decreased as treatment duration increased; (2) in alkaline pH at 35 °C, the concentration firstly decreased significantly but then recovered as treatment duration increased; (3) in acid and neutral pH at 55 °C, the concentration increased as treatment duration increased; and (4) in alkaline pH at 55 °C, a significant quantity of polysaccharides shifted from the TB fraction to supernatant initially and continued this shift as treatment duration increased. The use of polygalacturonase had extraordinary effects on the dissolution and redistribution of polysaccharides. The total content of polysaccharides of EPS fractions reached 7 times that of raw sludge. Although the polysaccharides content in TB did not increase appreciably, the content in supernatant, slime, and LB increased by as much as 50 % ± 6 % of the total polysaccharides content of raw sludge. In the cellulase treatment group at 55 °C, the polysaccharides content shifted significantly from TB to supernatant, while there was no evident increase in the total content of polysaccharides in EPS. DNase addition caused the polysaccharides content to increase in the TB fraction but did not affect the content of polysaccharides in other EPS fractions. The distribution of polysaccharides among EPS fractions of samples in the amylase group (in both the 35 and 55 °C treatments) showed little difference compared to the pH 6.9 control group. The proteinase group in the 55°C treatment showed a minor increase in the polysaccharides content of supernatant.

#### Redistribution of eDNA

The profile of EPS eDNA after the treatments followed four patterns: (1) in acid and neutral pH at 35 °C, the concentration increased slightly at first and then decreased sharply and remained low until the end of treatment; (2) in alkaline pH at 35 °C, the concentration firstly decreased significantly and then slowly recovered as treatment progressed, but remained low; (3) in acid pH at 55 °C, the total concentration and distribution of eDNA remained constant; and (4) in neutral and alkaline pH at 55 °C, a significant shift of eDNA occurred from the TB fraction to supernatant, slime, and LB fractions as treatment duration increased. Polygalacturonase (at pH 4.0) caused an approximately 7 % ± 2 % shift of eDNA from the TB fraction to supernatant, slime, and LB fractions at both 35 and 55 °C, but the total concentration of eDNA at 35 °C was higher than at 55 °C.

Amylase treatment (at pH 6.9) and proteinase treatment (at pH 6.9) affected eDNA distribution in similar ways. At 35 °C, both enzymes initially caused the eDNA concentration to increase significantly (compared to that in the raw sludge) to about 43 % of the total amount of sludge, but the concentration decreased to its initial value as treatment duration increased, while the distribution among the EPS fractions remained relatively constant throughout treatment. At 55 °C, amylase and proteinase caused significant increases in both the total amount of eDNA in EPS and the redistribution of eDNA from the TB to supernatant, slime, and LB fractions as treatment duration increased. The use of DNase (at pH 8.0) led to an initial decrease in the total amount of EPS, which slightly increased as treatment progressed, and although the distribution of eDNA in EPS did not change at 35 °C, eDNA gradually shifted to the supernatant and slime fractions at 55 °C. Cellulase (at pH 8.0) did not shift eDNA among the EPS fractions, but the total amount of eDNA was affected at 35 °C. At 55 °C, cellulase treatment significantly increased both the total amount and redistribution of eDNA as treatment duration increased.

An additional file presented that the significance values from ANOVA analysis revealed the differences among groups with the same group, so the effect of each enzyme is highlighted out of pH adjustment (see Additional file 2).

#### Redistribution of organics containing carbon

Compared to that in the pH 4.0 and pH 6.9 controls, DOC and DN in the pH 8.0 control was redistributed to a larger extent at both 35 and 55 °C, and the 55 °C treatment also resulted in an increase in the total concentrations. This effect of pH and temperature also applied to the enzyme addition samples. Amylase (at pH 6.9) and proteinase (at pH 6.9) treatments had very similar effects on DOC distribution; the two enzymes decreased the total amount of DOC in the 35 °C treatment and increased the DOC amount in the 55 °C, with greater redistribution among the fractions. Similarly, the DNase (at pH 8.0) and cellulase (at pH 8.0) treatments had similar effects on DOC, but to a much larger extent. The polygalacturonase treatment (at pH 4.0) caused changes in DOC that were different from those in the pH 4.0 control group; the total amount of DOC was not the smallest, and the extent of redistribution was rather high. And the 55 °C treatment only showed minor difference from the 35 °C treatment in terms of DOC redistribution.

#### Redistribution of organics containing nitrogen

The behavior of DN was very much like that of DOC, except that in the polygalacturonase treatment, DN changes corresponded strongly to those in the pH 4.0 control samples. Higher pH and temperature treatments seemed to promote greater DOC and DN redistribution, and enzyme addition had comparatively less impact. As the additional file presented, the calculated quantities of carbon contained in proteins (Fig. 1a), polysaccharides (Fig. 1b), and eDNA (Fig. 1c), and of nitrogen contained in proteins and eDNA were generally consistent with DOC (Fig. 1d) and DN profiles (Fig. 1e) (see Additional file 3).

#### PCA analysis on organic distribution profiles

**Fig. 2:**
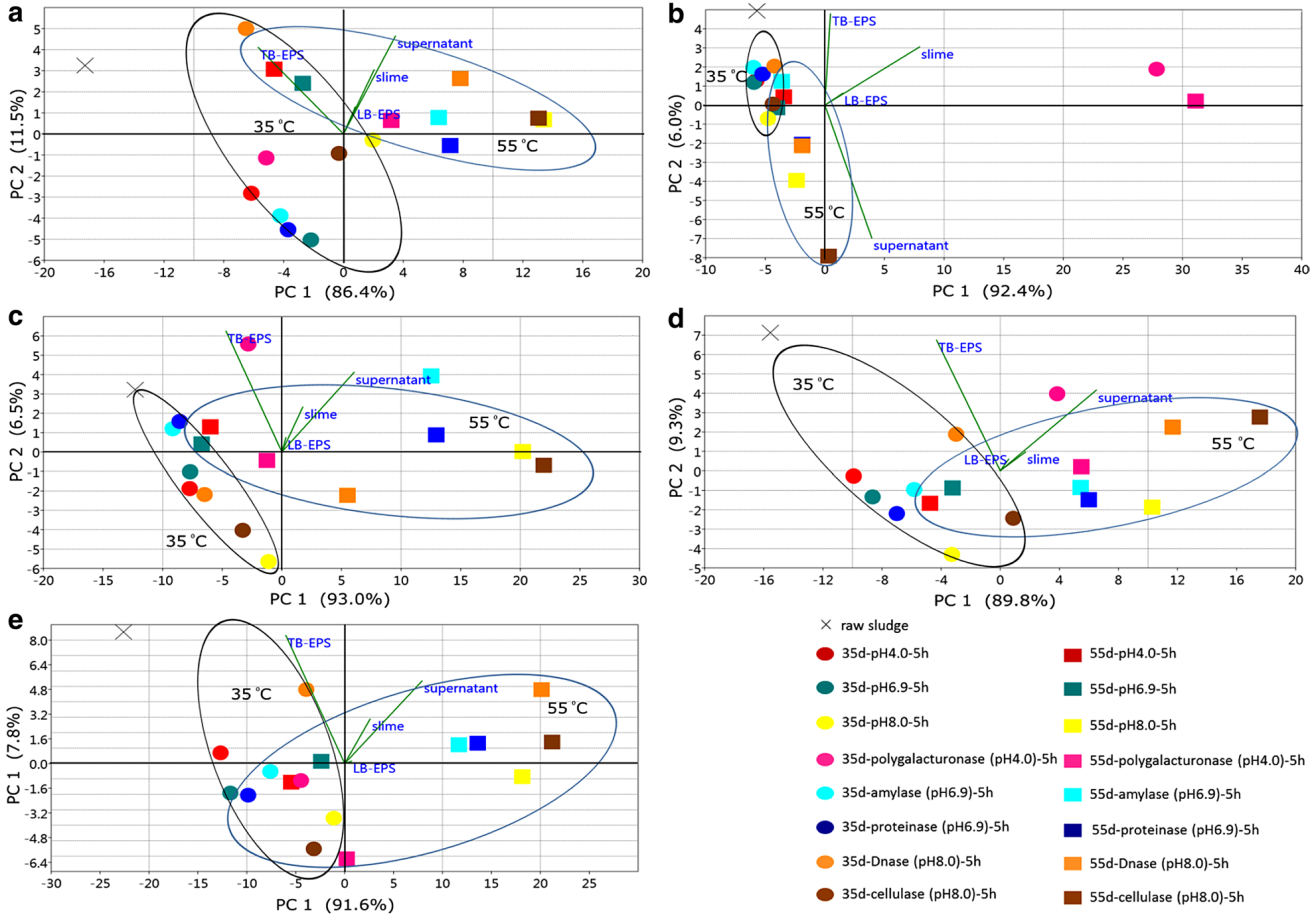
PCA results of organic matters in different fractions. **a** EPS protein, **b** EPS polysaccharides, **c** EPS DNA, **d** EPS carbon, **e** EPS nitrogen

PCA results on each indicator for organics are shown in Fig. 2, which obviously observed the different effect of diverse treatments. From Fig. 2a–e, that the vector of “supernatant” is orthogonal to the vector “TB-EPS,” which represents totally different distribution levels of organic matters. Temperature showed strong impact on EPS fractions distributing to supernatant and TB-EPS, with samples under 35 °C treatments close to “TB-EPS,” and samples under 55 °C treatments closer to “supernatant.” And samples showed patterns to gather according to different pH adjustments, too. Basically, the relative position of samples in the PCA results acted in coordination with content analysis above, especially on alkaline environment improving organic matters’ release. Moreover, polygalacturonase’s improvement on polysaccharides distribution to supernatant and cellulase promoting most kinds of organic matters release from TB-EPS are quite clear.

### Redistribution of dissolved fluorescent substances

**Fig. 3:**
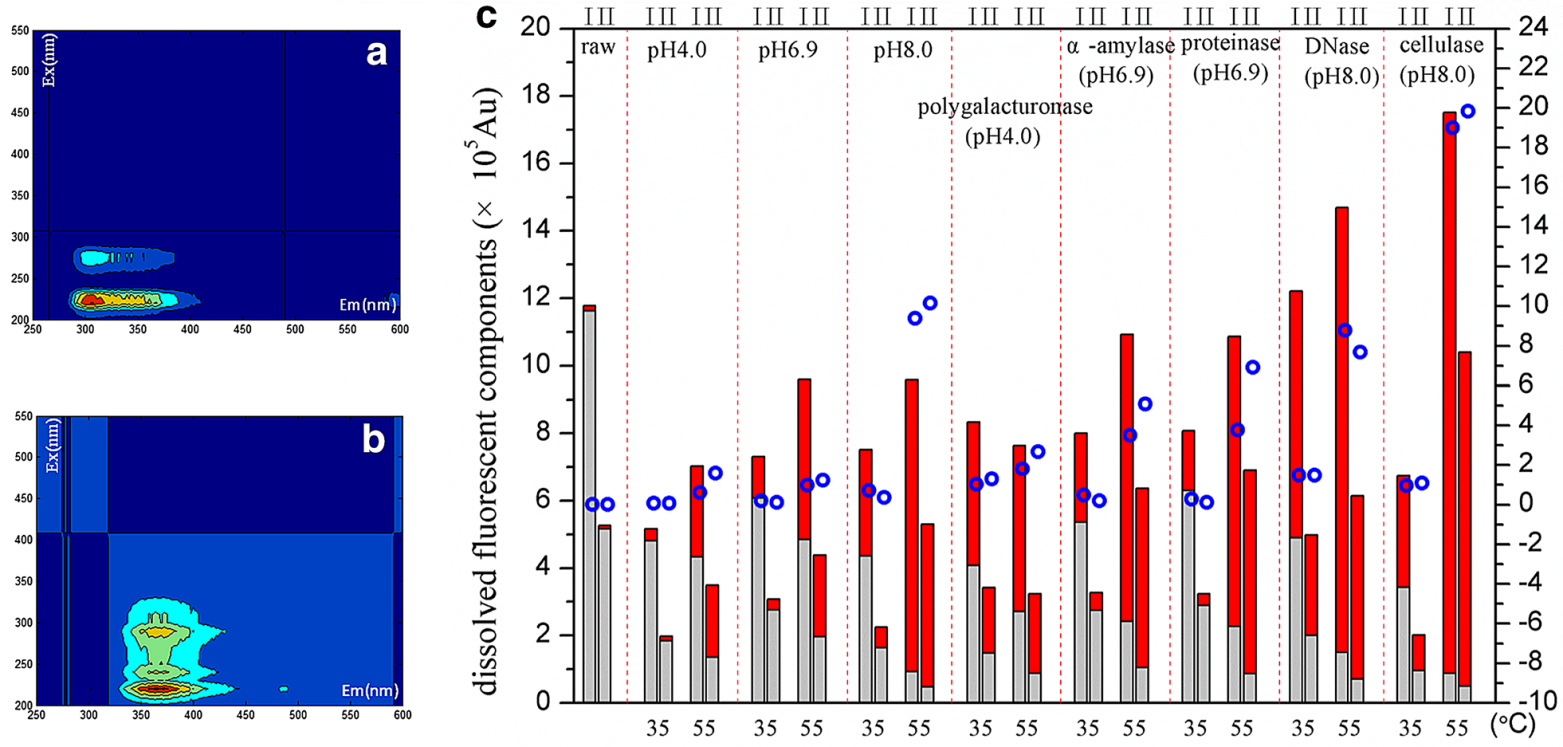
EEM results. **a** Component I, **b** Component II, **c** distribution of dissolved fluorescent components. *Red square* supernatant, *gray square* tightly bound; *blue open circle* ratio of supernatant to tightly bound

Fluorescent excitation–emission matrix analysis (EEM) was used to understand the extracellular distribution of recalcitrant organic matter (e.g., humic substances) which might contribute to the EPS biodegradability or dewaterability. The PARAFAC analysis of EEM data extracted two components that contributed 90.6 % of spectrum data variation. Component I, which peaked at excitation (Ex) wavelengths of 220/280 nm and emission (Em) wavelengths of 306/356 nm (Fig. 3a), represented proteins and soluble microbial products. Component II, which peaked at Ex 220/240/290 nm and Em 364 nm (Fig. 3b), represented organic substances that had a higher degree of humification, according to the classification by Chen et al. [20] and Lü et al. [19]. The distribution of fluorescent substances is shown in Fig. 3c. It shows that the score of Component I was twice that of Component II in the amount among the groups, including raw sludge. In raw sludge, fluorescent substances were mostly distributed in the TB-EPS fraction and rarely in supernatant. The concentration ratio of Component I substances in supernatant to those in the TB-EPS fraction increased from 0.014 in raw sludge to 1.4 and 19.0 following the 35 and 55 °C treatments, respectively. Similarly, the ratio of Component II substances in the supernatant to those in the TB-EPS fraction increased from 0.02 (raw sludge) to 1.48 and 19.8 following treatment at 35 and 55 °C, respectively. Both the extent of redistribution and the total content of each component in supernatant were greater under the 55 treatment than in the 35 °C treatment. Like temperature, pH also played a role in the redistribution of fluorescent substances. The total content and extent of redistribution into supernatant were quite low in the pH 4.0 control group, but both measures increased in the pH 6.9 control group and increased further in the pH 8.0 control group.

In the polygalacturonase treatment (at pH 4.0), the two treatment temperatures (35 and 55 °C) had very similar effects on the total content and redistribution of fluorescent substances in the two EPS fractions. The amylase (at pH 6.9) and proteinase (at pH 6.9) enzymes had similar effects. Compared to raw sludge, both enzymes resulted in a lower total content of the substances in the TB-EPS, but a greater redistribution to supernatant under the 35 °C treatment, and almost the same total content and an even greater redistribution under the 55 °C treatment. However, in the 35 °C treatment, amylase and proteinase caused the lowest redistribution of fluorescent substances from the TB-EPS fraction to supernatant among all enzymatic treatments. The DNase treatment (at pH 8.0 and 35 °C) produced the greatest increases in the total content of fluorescent substances and in the redistribution of both components of fluorescent substances. At 35 °C, the cellulase treatment (at pH 8.0) produced changes like those in the pH 8.0 control group; however, at 55 °C cellulase increased, the total content of Component I and II substances by 48 and 100 %, respectively, compared to the content in raw sludge. In addition, among all enzyme treatments, cellulase caused the highest migration of fluorescent substances from the TB-EPS fraction to supernatant.

### Morphology changes of pellets

**Fig. 4:**
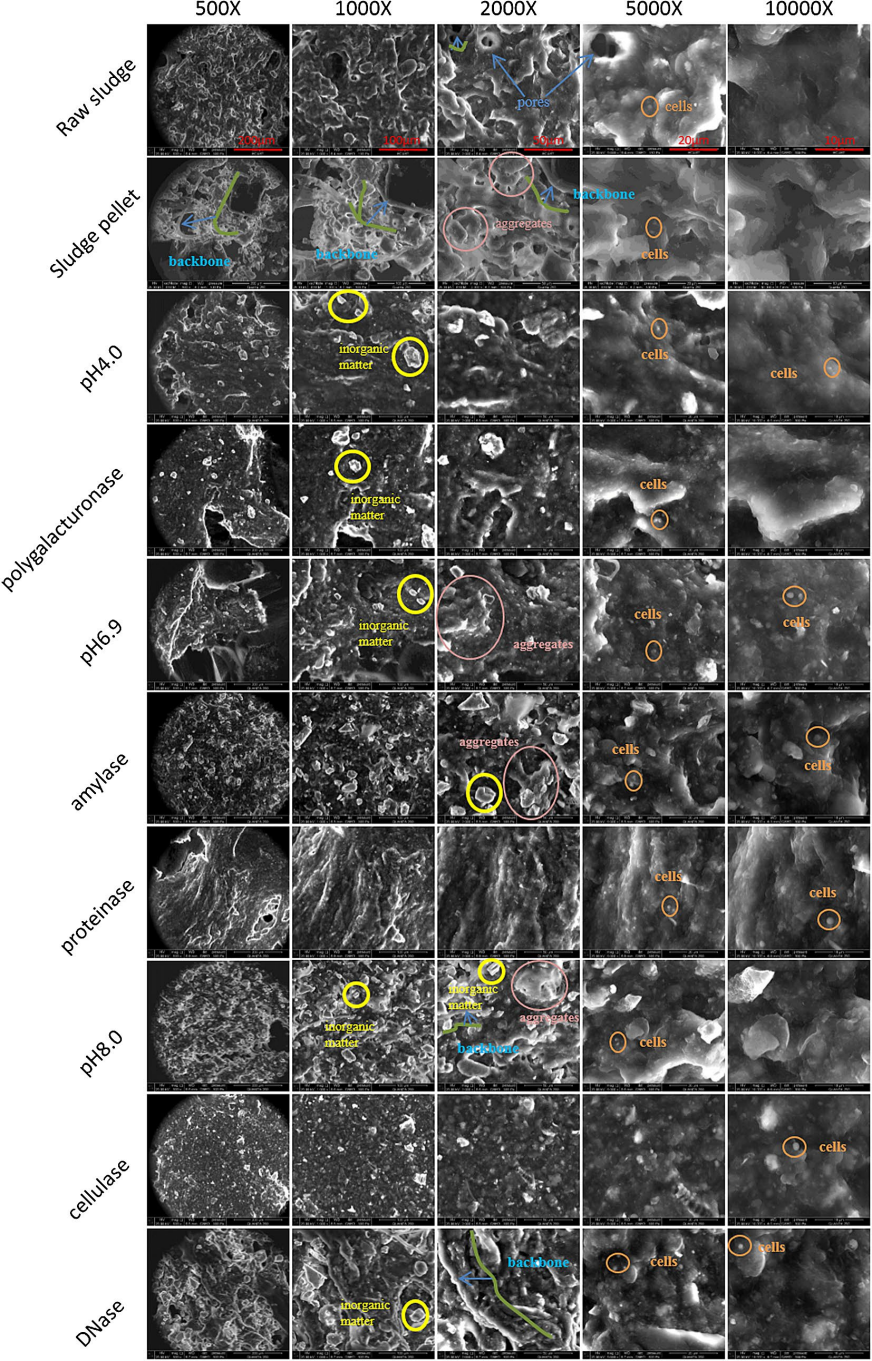
ESEM results (×500, ×1000, ×2000, ×5000, ×10,000)

Scanning electron microscopy was used to visualize the morphology of raw sludge and pellets after different treatments; the ESEM images are shown in Fig. 4. These images show differences between the microstructures of raw sludge and those of the pellets resulting from various treatments. Both raw sludge and the pellets demonstrated a rich spatial structure composed of porous “backbones,” abundant EPS flocs, and thick superficial microbes, similar to what was reported by [21].

After enzymatic treatment and pH adjustment, a massive number of free particles were observed in samples. And the surficial microstructure abundance decreased more or less, especially in samples to which polygalacturonase, proteinase, and cellulase had been added. In the ESEM pictures of these three enzyme groups, “backbone,” inorganic particle, and aggregate are seldom observed, and they appeared plainer and more homogenous. The pores inside individual particles became more dispersed and smaller. These structural changes could have reflected EPS disintegration. In fact, different treatments had distinctive morphological effects, which indicated different spatial distributions or roles of the hydrolyzed substrates in EPS formation.

Four types of changes in microstructure were observed. The first type of microstructure change was associated with the pH 6.9 treatment, pH 4.0 treatment, and polygalacturonase treatment (at pH 4.0) in which the surface of the bulk raw sludge appeared smoother and less abundant of arches and filamentous structures than did the sludge pellet. The second type of microstructure change resulted from amylase treatment (at pH 6.9), pH 8.0 treatment, and DNase treatment (at pH 8.0). The whole field of vision in ESEM images displayed rich superficial microstructures similar to the sludge pellet observation, but no apparent backbone could be observed. The third type of microstructure change was visible in the cellulase treatment (at pH 8.0). This type of change was characterized by a much plainer and simpler structure with only an accumulation of granules. The fourth type of microstructure change resulted from the proteinase treatment (at pH 6.9). The most distinctive feature of samples in this treatment was a vertical spatial structure, similar to that of a cave with inclined sleek walls tier upon tier.

From results observed in ESEM, some groups with different pH had similar morphology changes. It is clear that these differences did not caused by pH changes. Hence, the specific effect of each enzyme led to diverse types of EPS disintegration.

### Dewaterability of treated sludge

**Fig. 5:**
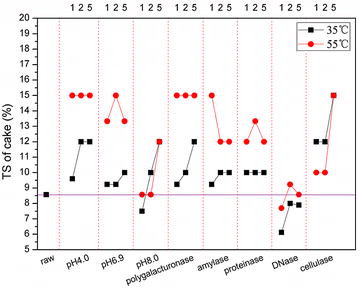
TS of cakes after centrifugation at 2000×*g* for 10 min

The TS content of cake from raw sludge was 8.5 % wt and increased after treatment (Fig. 5). Within any treatment group, sludge that was treated at 55 °C showed higher dewaterability than when treated at 35 °C. Furthermore, cake TS decreased as pH increased. The TS of cake from pH 4.0 control samples and from polygalacturonase (at pH 4.0) treatment samples reached 15 % wt (the highest of all samples) quickly and remained high for all treatment durations, while the TS in cake from other treatments started at lower concentrations and increased as the duration of treatment increased. At the end of treatment, most treatments produced cakes that had a TS content over 12 %wt in the 55 °C treatment and over 10 %wt in the 35 °C treatment. In contrast, the TS content of cake from the DNase treatment decreased significantly to 6 %wt, followed by increasing dewaterability; however, the dewaterability of DNase-treated sludge was not better than that of raw sludge.

### Role of temperature, pH and different hydrolases

In this study, the quantity of organic substances released from the interior fraction (TB-EPS) of activated sludge to the exterior fraction (supernatant) was greater at higher pH and higher temperature, even without added enzymes. These results demonstrate that activated sludge flocs are very unstable in an alkaline environment and at high temperature (i.e., 55 °C). Alkaline environment was widely applied to improve sludge’s hydrolysis degree [14, 15], and it is reported that higher temperature could promote the abiotic dissolution of proteins [22]. And higher temperature could speed up the enzymatic hydrolysis so as to demonstrate larger impact on disintegrating sludge floc.

As shown in Figs. 1, 2 and 3, enzymes affected the release of organic matter from the inner fraction (TB-EPS) to the most exterior fraction (supernatant) to different extents. Polygalacturonase was especially effective in mobilizing polysaccharides, with increases up to 700 % in total EPS polysaccharides amount, compared with raw sludge. However, proteins and DNA were not released in large amounts in accordance with polysaccharides, which meant these three components were not from the same source (e.g., resulted from bacteria cell lysis). Hence, it is unlikely that massive polysaccharides came out of cells lysis. Furthermore, the DN content of sludge treated using polygalacturonase was released to a much less extent than by other enzyme treatments, and the C/N ratio of polygalacturonase-treated samples decreased as incubation time increased. Combined with the ESEM results, which in this treatment were slightly different from those from the sludge pellet, the analytical results indicate that the polygalacturonic acid hydrolyzed by polygalacturonase has an important role in EPS floc formation (i.e., linkages of small flocs) but has no direct links with proteins or other organic matter that would be liberated when the linkages are broken. Hence, under polygalacturonase treatment, the polysaccharides content of sludge changed hugely, yet without a massive release of proteins or eDNA. Furthermore, compared with the ESEM observation of the pH 6.9 control group, there was no obvious relationship with the surfaces of floc from the polygalacturonase-treated sludge, suggesting the substrate that is acted upon by polygalacturonase is too minor to introduce significant morphological change.

Cellulase treatment caused the release of all organic species at a relatively high level, as indicated by DOC (Fig. 1d), DN (Fig. 1e), and humic-like substances (Fig. 3). Since cellulase acts on cellulose, or the so-called beta-carbohydrate, which comprises 1.2–38.9 %dw [23] of active sludge, the plain microstructure may indicate that beta-carbohydrate is the adhesive of the organic matter within the floc. When this adhesive was hydrolyzed by cellulase, the floc was dramatically broken down. As a result, the ESEM image of cellulose-treated sludge (Fig. 4) revealed a homogenous appearance that lacked structure.

### A proposed hypothesis on EPS biochemical distribution model

Many conceptual models have been developed to elucidate the biological flocculation process and compositional distribution in the floc. The existing conceptual models can be classified into two types. In the first type, the whole floc is considered to be a gel-like matrix containing cell particles and organic colloids homogeneously bridged by various polymers and usually described using the Sierpinski carpet model [24–28]. In contrast, the second type of model considers the spatial distribution of biomass cells to be inhomogeneous, an interpretation mostly concluded from mathematical simulation of sludge floc growth, calculated parameters such as porosity, and morphological changes [29, 30]. Proteins, polysaccharides, and glycoproteins are considered to be the adhesive substances among microbial cells or between cells and attachable particles in the floc-forming process and are considered to be capable of influencing the subsequent treatable characteristics of activated sludge. These structural models normally consider the integrity of individual microbial cells or exopolymer particulates but rarely specifically describe the inner organic composition of cells and particulates; in addition, floc structures are usually described using particle size and porosity to distinguish among cells, fibers, and exopolymer.

However, asynchronous migration of carbon- and nitrogen-containing matter was observed in the present enzymatic study, as well as the unexpected hydrolysis efficiency of different enzymes. For example, polygalacturonase treatment changed the polysaccharides content of EPS significantly, yet without massive release of proteins or eDNA. The polysaccharide-releasing efficiency of polygalacturonase was far higher than that of the carbohydrases targeting amylum and cellulose, which were regarded as the major polysaccharides in EPS [5, 6]. In addition, the proteinase that targeted proteins, which comprised the majority of EPS organic material, was almost incapable of releasing protein; instead, DNase showed higher protein-releasing efficiency.

Therefore, the various objective polymers (e.g., proteins and polysaccharides) must have different spatial distributions within EPS. Much research on floc formation [5, 6, 31] has accepted that cellulose might be the backbone for the whole floc structure. In the present study, when cellulose was hydrolyzed by cellulase, both proteins and polysaccharides appeared in very high amounts in all treatment conditions and were highly redistributed among EPS fractions. These results indicated that cellulose is the constructive substance for EPS, such that when this “backbone” was destroyed, the organic contents of the sludge were readily released.

However, the role of polygalacturonase cannot be discounted. Polygalacturonase is an enzyme that hydrolyses the alpha-1, 4 glycosidic bonds between galacturonic acid residues. Galacturonic acid originating from bacterial source, as reported in the references [32, 33], is a component of bacterial polysaccharide and is important for cell structure. Frirdich and Whitfield [32] pointed out that *Klebsiella pneumoniae* l contains galacturonic acid residues that are proposed to serve a function in outer membrane stability. Ugalde et al [33] pointed out that a cell wall polymer containing galactose and galacturonic acid affects the bacterial strain’s agglutinability with lectin and virus sensitivity. Hence, it is quite possible that polygalacturonic acid-like matter becomes a component of the adhesives that bind sludge floc. The elimination of polygalacturonic acid-like matter could result in the disintegration of the whole extracellular polymer substances matrix. As confirmed in the present study, when polygalacturonic acid-like matter was hydrolyzed, the content of polysaccharides in supernatant soared up extremely high, while the content of proteins did not increase (Fig. 1). Furthermore, polygalacturonase will not only target on the polygalacturonic acid of bacterial source abundant in sludge floc, but it can also act on the polygalacturon of plant source, i.e., pectin. Since municipal wastewater treatment plant accepts sewage containing plant fiber [34], the recalcitrantly biodegradable pectin in sewage will finally accumulate in sludge floc. As a result, the added polygalacturonase can also take some effect by acting on this component.

**Fig. 6:**
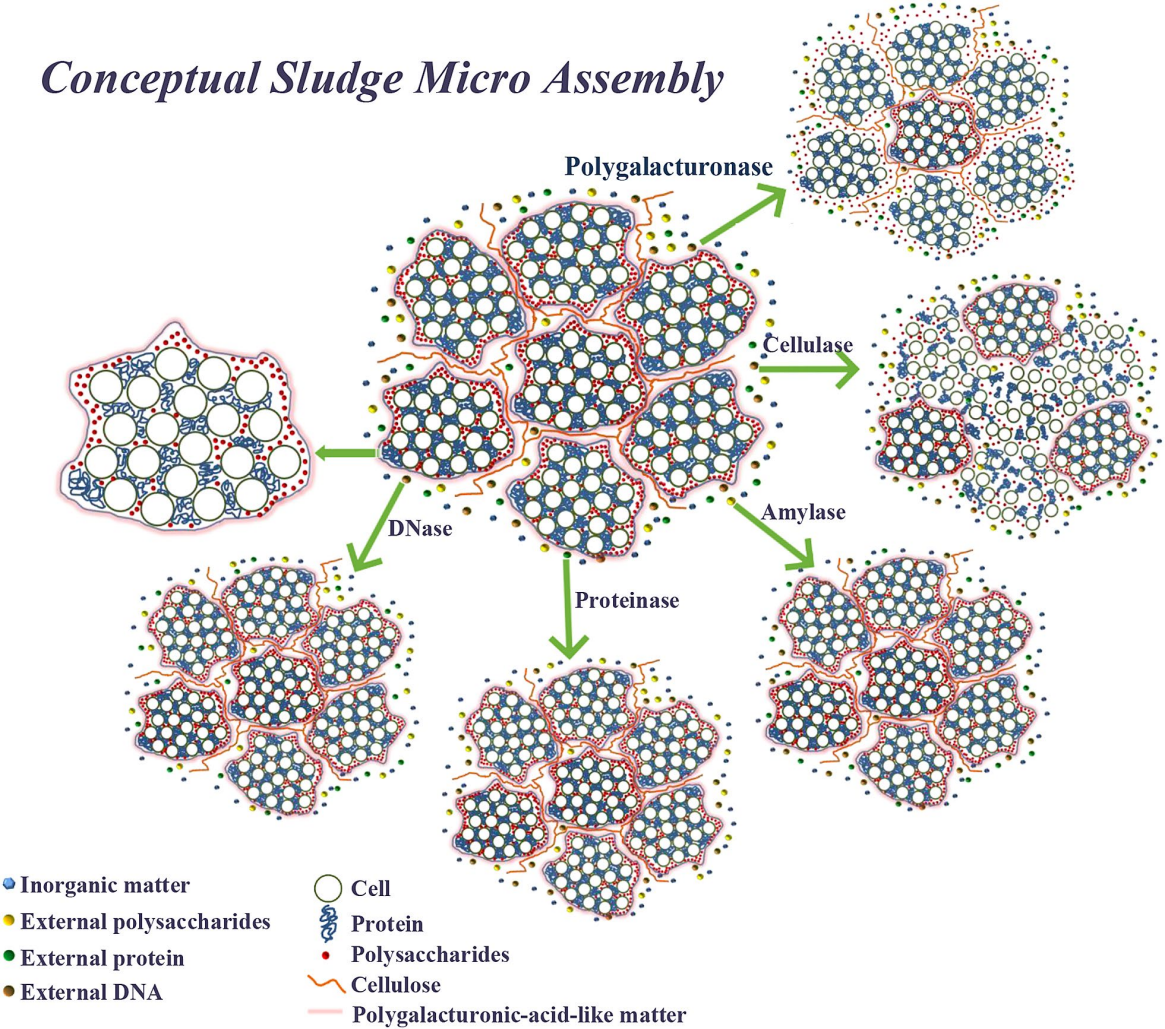
Hypothesis model about biochemical structure of EPS. *Note*: Five *green arrows* with text means treatments with enzymes, the *left arrow* without text points to the exaggerated micro-assembly

Hence, a plausible description of EPS (illustrated in Fig. 6) is that it consists of a large amount of small flocs in which major microbes and most proteins and other nitrogen-containing substances are firmly embedded. In addition, polygalacturonic acid-like matter might be a minor, but key, adhesive among small flocs, such that several small flocs together with fiber made of cellulose and other polysaccharides further construct the EPS. As a result, when polygalacturonic acid-like matter is hydrolyzed by polygalacturonase, cellulose and other polysaccharides are liberated, while proteins remain in the small flocs. If proteinase cannot access the protein aggregates and cells in small flocs easily, its efficiency in releasing proteins from sludge will be limited.

Nonetheless, the conceptual model needs experimental evidence to directly visualize the spatial distribution of exopolymers at nanometer scale. This requirement highlights the utility of high magnification microscopy combined with fluorescent probing or functional group probing techniques to revisit the structure of cell exopolymer, so as to better understand the relationship between sludge biochemical properties and sludge treatability. Currently, there are few published studies on the absolute content of polygalacturonic acid-like matter in sludge and EPS. Hence, the need is acute for research on this special minor organic species, as well as for an accurate determination method for the sludge matrix.

## Conclusions

Although being minor, polygalacturonic acid-like matter and cellulose were observed to be key substances on the disintegration of sludge, according to the results of enzymatic treatment in this study. Hence, their roles in the structure and formation of EPS were proposed in a hypothesis model of sludge. As indicated in results, polygalacturonase and cellulase can be used to improve digestion and dewaterability of sludge. On the basis of single enzyme’s effect, we suggest combinations of enzymes and multiple sludge sources be applied for further research.

## Additional files

10.1186/s13068-016-0444-y Procedure about the extraction of EPS fractions.
10.1186/s13068-016-0444-y ANOVA analysis of polysaccharides, protein and eDNA contents in supernatant and TB-EPS. In order to distinguish the effect of control group and experiment group at the same pH, ANOVA analysis was applied on the contents of polysaccharides, protein and eDNA in supernatant and TB-EPS fraction. pH 4.0 group includes pH 4.0 control and pectinase-added group; pH 6.9 group includes pH 6.9 control, amylase-added group and proteinase-added group; pH 8.0 group includes pH 8.0 control, cellulase-added group and DNase-added group. Supernatant and TB-EPS are the two representatives of the four fractions. The P-values of each group (pH 4.0, pH 6.9 and pH 8.0) are presented.
10.1186/s13068-016-0444-y Calculation of carbon and nitrogen contained in organics. The obtained values on protein, polysaccharide and DNA in EPS were converted into the basis of carbon or nitrogen, according to their molecular formula. Accordingly, the equations for carbon calculation and nitrogen calculation were listed. The purpose of calculation was to compare with the directly measured values of DOC and DN. The calculation results are shown.

## Abbreviations

EPS: extracellular polymeric substances
LB-EPS: loosely bound EPS
TB-EPS: tightly bound EPS
TS: total solids
VS: volatile solids
DOC: dissolved organic carbon
DN: dissolved nitrogen
eDNA: external DNA
EEM: excitation–emission matrix
PARAFAC: parallel factor analysis
ESEM: environmental scanning electron microscope
PCA: principal component analysis
