# Supplementary material for: Enzyme disintegration with spatial resolution reveals different distributions of sludge extracellular polymer substances
Source: Biotechnol Biofuels. 2016 Feb 3;9:29. doi: 10.1186/s13068-016-0444-y (PMC4739380; doi:10.1186/s13068-016-0444-y)
Supplement: Supplementary file 1 — 10.1186/s13068-016-0444-y Procedure about the extraction of EPS fractions. [file 13068_2016_444_MOESM1_ESM.docx]

**Additional file 1: Procedure about the extraction of EPS fractions**

The extraction procedure suggested by Yu et al. (2008) was followed with some modification. Briefly, the sludge samples were first centrifuged at 2,000×g for 10 min, and the resulting bulk solution was called the supernatant fraction. Then, the residues were re-suspended to the initial volume using phosphate buffer solution (containing 8.36 mM NaH_2_PO_4_, 0.137 mM NaCl, 2.7 mM KCl, 1.47 mM KH_2_PO_4_, pH 7) followed by centrifuging at 5,000×g for 10 min, and the resulting bulk solution was collected as the slime fraction. Next, the residues were re-suspended as before and centrifuged at 5,000×g for 10 min. The resulting bulk solution was collected as the LB-EPS. Following retrieval of LB-EPS, the remaining residues were re-suspended to the initial volume for the final time, and treated by ultrasonication at 20 kHz and 480 W for 10 min, followed by centrifugation at 12,000×g for 10 min. The resulting bulk solution was collected as the TB-EPS and the solid phase residual was collected as the pellet.
