# Supplementary material for: Enzyme disintegration with spatial resolution reveals different distributions of sludge extracellular polymer substances
Source: Biotechnol Biofuels. 2016 Feb 3;9:29. doi: 10.1186/s13068-016-0444-y (PMC4739380; doi:10.1186/s13068-016-0444-y)
Supplement: Supplementary file 2 — 10.1186/s13068-016-0444-y ANOVA analysis of polysaccharides, protein and eDNA contents in supernatant and TB-EPS. In order to distinguish the effect of control group and experiment group at the same pH, ANOVA analysis was applied on the contents of polysaccharides, protein and eDNA in supernatant and TB-EPS fraction. pH 4.0 group includes pH 4.0 control and pectinase-added group; pH 6.9 group includes pH 6.9 control, amylase-added group and proteinase-added group; pH 8.0 group includes pH 8.0 control, cellulase-added group and DNase-added group. Supernatant and TB-EPS are the two representatives of the four fractions. The P-values of each group (pH 4.0, pH 6.9 and pH 8.0) are presented. [file 13068_2016_444_MOESM2_ESM.docx]

**Additional file 2: ANNOVA analysis of polysaccharides, protein and eDNA contents in supernatant and TB-EPS**

In order to distinguish the effect of control group and experiment group at the same pH, ANNOVA analysis was applied on the contents of polysaccharides, protein and eDNA in supernatant and TB-EPS fraction. pH4.0 group includes pH4.0 control and pectinase-added group; pH6.9 group includes pH6.9 control, amylase-added group and proteinase-added group; pH8.0 group includes pH8.0 control, cellulase-added group and DNase-added group. Supernatant and TB-EPS are the two representatives of the four fractions. The P-values of each group(pH4.0, pH6.9 and pH8.0) are shown below.

**Table. A1 ANNOVA analysis of organic matters content**

| **group** | **T(⁰C)** | **T(h)** | **Polysaccharides** | | **Protein** | | **eDNA** | |
| --- | --- | --- | --- | --- | --- | --- | --- | --- |
|  |  |  | **supernatant** | **TB-EPS** | **supernatant** | **TB-EPS** | **supernatant** | **TB-EPS** |
| **pH4.0** | **35** | **1** | 0.00043 | 8.53E-07 | 2.05E-05 | 0.000134 | 0.583631 | 0.757309 |
|  |  | **2** | 0.013162 | 2.18E-05 | 0.001609 | 0.566049 | 6.65E-06 | 0.087115 |
|  |  | **5** | 1.1E-06 | 0.000532 | 7.1E-07 | 0.861459 | 1.43E-06 | 0.187998 |
|  | **55** | **1** | 2.6E-11 | 0.127674 | 1.23E-07 | 0.033228 | 0.001874 | 0.17236 |
|  |  | **2** | 1.81E-07 | 0.673075 | 1.81E-05 | 3.19E-06 | 0.000166 | 0.335836 |
|  |  | **5** | 8.56E-06 | 0.007434 | 5.36E-05 | 0.000515 | 0.006113 | 0.006135 |
| **pH6.9** | **35** | **1** | 3.9E-06 | 0.775013 | 0.001034 | 0.049012 | 0.001176 | 0.000122 |
|  |  | **2** | 4.63E-08 | 0.141337 | 0.000815 | 0.007588 | 5.68E-05 | 0.059692 |
|  |  | **5** | 8.61E-05 | 0.230652 | 5.78E-05 | 0.000554 | 0.000292 | 0.05802 |
|  | **55** | **1** | 6.96E-07 | 3.02E-05 | 4.04E-07 | 7.2E-06 | 0.001087 | 3.59E-06 |
|  |  | **2** | 3.59E-05 | 3.81E-06 | 8.58E-10 | 7.54E-06 | 1.66E-10 | 0.000661 |
|  |  | **5** | 3.74E-10 | 0.000622 | 2.67E-09 | 3.59E-11 | 7.75E-07 | 6.17E-06 |
| **pH8.0** | **35** | **1** | 4.25E-07 | 3.54E-06 | 1.94E-05 | 4.86E-07 | 0.10729 | 0.000193 |
|  |  | **2** | 5.65E-08 | 5.64E-05 | 6.82E-07 | 1.09E-09 | 0.205908 | 0.000159 |
|  |  | **5** | 0.000175 | 0.000409 | 2.54E-05 | 2.14E-06 | 0.50924 | 0.114767 |
|  | **55** | **1** | 1.29E-08 | 3.2E-08 | 0.000117 | 1.75E-10 | 0.105419 | 0.006211 |
|  |  | **2** | 3.21E-05 | 2.78E-07 | 0.05688 | 3.72E-07 | 0.61909 | 0.0026 |
|  |  | **5** | 0.002189 | 7.56E-08 | 0.414768 | 0.002678 | 0.926649 | 0.004901 |

Note: red colored number means P>0.05.
