# Supplementary material for: Enzyme disintegration with spatial resolution reveals different distributions of sludge extracellular polymer substances
Source: Biotechnol Biofuels. 2016 Feb 3;9:29. doi: 10.1186/s13068-016-0444-y (PMC4739380; doi:10.1186/s13068-016-0444-y)
Supplement: Supplementary file 3 — 10.1186/s13068-016-0444-y Calculation of carbon and nitrogen contained in organics. The obtained values on protein, polysaccharide and DNA in EPS were converted into the basis of carbon or nitrogen, according to their molecular formula. Accordingly, the equations for carbon calculation and nitrogen calculation were listed. The purpose of calculation was to compare with the directly measured values of DOC and DN. The calculation results are shown. [file 13068_2016_444_MOESM3_ESM.docx]

**Additional file 3: Calculation of carbon and nitrogen contained in organics**

The obtained values on protein (**Fig.1**), polysaccharide (**Fig.2**) and DNA (**Fig.3**) in EPS were converted into the basis of carbon or nitrogen, according to their molecular formula, i.e. C_5_H_7_O_2_N for protein, C_6_H_12_O_6_ for polysaccharide and C_19_H_21_N_15_O_4_ for DNA [referring to E. Coli DNA, (Blattner et al. 1997)]. Accordingly, the equations for carbon calculation and nitrogen calculation were listed as **Eq. (A.1)** and **Eq. (A.2)**. The purpose of calculation was to compare with the directly measured values of DOC and DN. The calculation results are shown in **Fig. (A.1)** and **Fig. (A.2)**.

Calculated carbon content (μg) = polysaccharides content (μg) × 72/180 + protein content (μg) × 60/113+ DNA content (μg) × 57/126.7 (**Eq.A.1**)

Calculated nitrogen content (μg) = protein content (μg) = protein content (ontμg) ×52.5/126.7 (**Eq.A.2**)


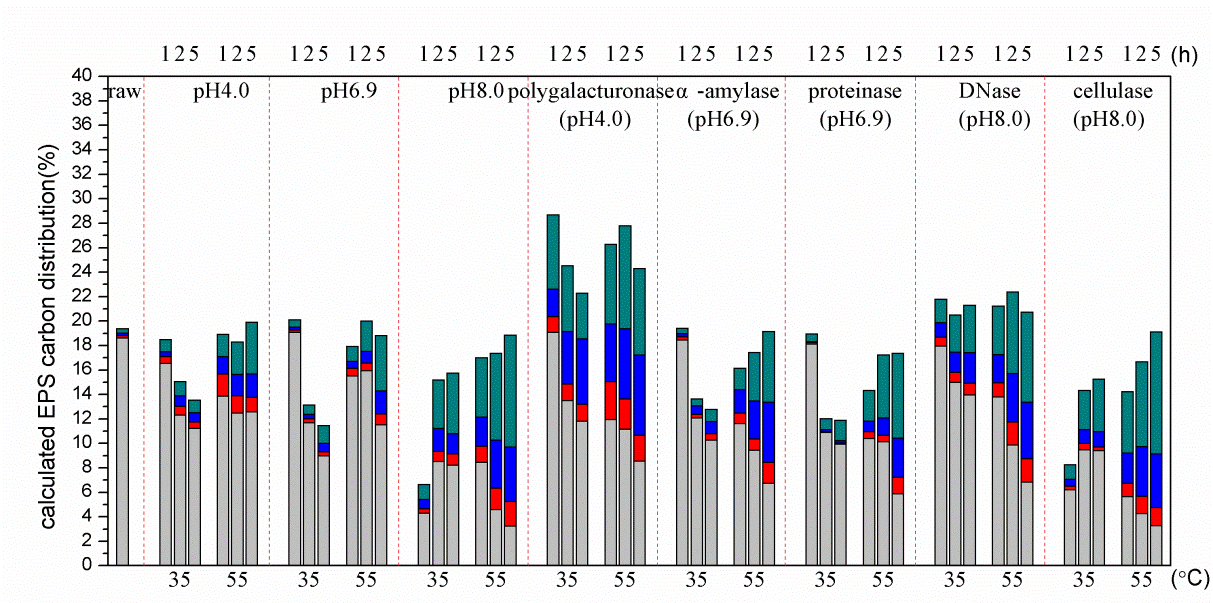


**Fig. A.1** Calculated carbon distribution.


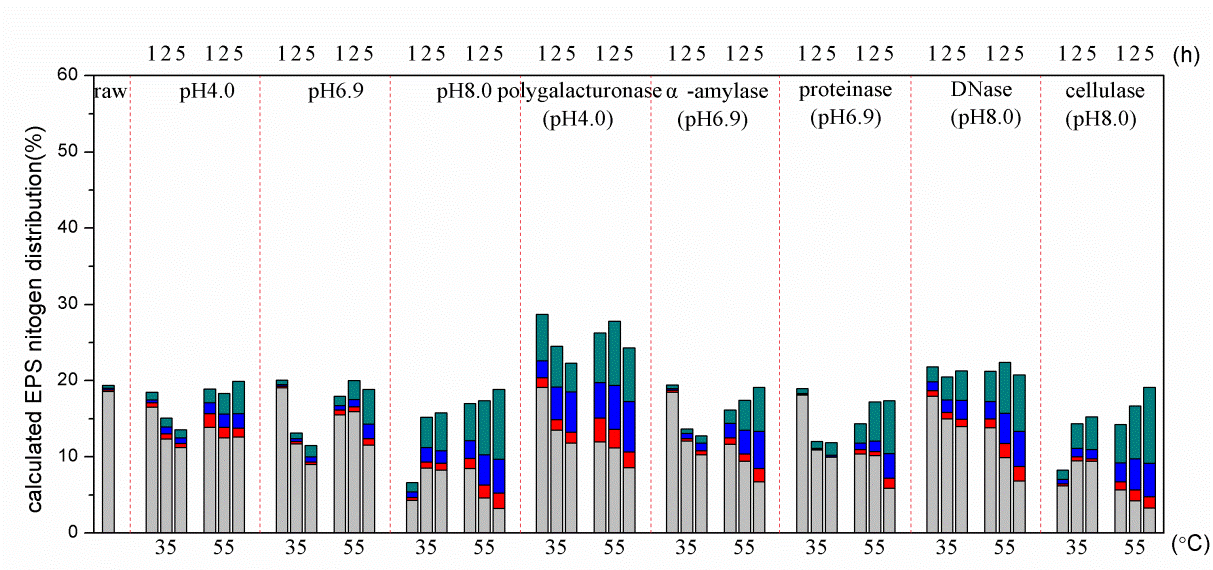


**Fig.A.2** Calculated nitrogen distribution.

**Literature Cited**

Yu, G.-H., He, P.-J., Shao, L.-M. and Zhu, Y.-S. (2008) Extracellular proteins, polysaccharides and enzymes impact on sludge aerobic digestion after ultrasonic pretreatment. Water Research 42(8–9), 1925-1934.

Blattner, F.R., Plunkett, G., Bloch, C.A., Perna, N.T., Burland, V., Riley, M., Collado-Vides, J., Glasner, J.D., Rode, C.K., Mayhew, G.F., Gregor, J., Davis, N.W., Kirkpatrick, H.A., Goeden, M.A., Rose, D.J., Mau, B. and Shao, Y. (1997) The Complete Genome Sequence of Escherichia coli K-12. Science 277(5331), 1453-1462.
